# Qualitative analysis of how patients decide that they want risk-reducing mastectomy, and the implications for surgeons in responding to emotionally-motivated patient requests

**DOI:** 10.1371/journal.pone.0178392

**Published:** 2017-05-26

**Authors:** Stephen L. Brown, Demian Whiting, Hannah G. Fielden, Pooja Saini, Helen Beesley, Christopher Holcombe, Susan Holcombe, Lyn Greenhalgh, Louise Fairburn, Peter Salmon

**Affiliations:** 1 Department of Psychological Sciences, University of Liverpool, Liverpool, United Kingdom; 2 Hull York Medical School, Hull, United Kingdom; 3 Royal Liverpool and Broadgreen University Hospitals NHS Trust, Liverpool, United Kingdom; 4 Royal Liverpool Women’s Hospital NHS Trust, Liverpool, United Kingdom; Tata Memorial Centre, INDIA

## Abstract

**Objective:**

Contemporary approaches to medical decision-making advise that clinicians should respect patients’ decisions. However, patients’ decisions are often shaped by heuristics, such as being guided by emotion, rather than by objective risk and benefit. Risk-reducing mastectomy (RRM) decisions focus this dilemma sharply. RRM reduces breast cancer (BC) risk, but is invasive and can have iatrogenic consequences. Previous evidence suggests that emotion guides patients’ decision-making about RRM. We interviewed patients to better understand how they made decisions about RRM, using findings to consider how clinicians could ethically respond to their decisions.

**Methods:**

Qualitative face-to-face interviews with 34 patients listed for RRM surgery and two who had decided against RRM.

**Results:**

Patients generally did not use objective risk estimates or, indeed, consider risks and benefits of RRM. Instead emotions guided their decisions: they chose RRM because they feared BC and wanted to do ‘all they could’ to prevent it. Most therefore perceived RRM to be the ‘obvious’ option and made the decision easily. However, many recounted extensive post-decisional deliberation, generally directed towards justifying the original decision. A few patients deliberated before the decision because fears of surgery counterbalanced those of BC.

**Conclusion:**

Patients seeking RRM were motivated by fear of BC, and the need to avoid potential regret for not doing all they could to prevent it. We suggest that choices such as that for RRM, which are made emotionally, can be respected as autonomous decisions, provided patients have considered risks and benefits. Drawing on psychological theory about how people *do* make decisions, as well as normative views of how they *should*, we propose that practitioners can guide consideration of risks and benefits even, where necessary, after patients have opted for surgery. This model of practice could be extended to other medical decisions that are influenced by patients’ emotions.

## Introduction

Current normative views of medical decision-making exhort clinicians to respect patients’ preferences and to be guided by them when making treatment decisions [1]. This approach assumes that patients’ preferences reflect ‘rational’ choices; that is, they have deliberated about decisions, looked at and understood the evidence, and weighed the options available and their respective risks and benefits [2].Yet, patients often lack sufficient understanding of clinical issues or feel too distressed to think carefully about decisions [3]. Thus, they often use reasoning ‘short-cuts’, or ‘heuristics’, to make decisions [4,5]. How clinicians should respond to heuristically-based decisions is unclear. Reasoning that heuristics can introduce inaccuracy or bias, it has been suggested that such decisions should carry less weight than those made rationally because they may distort decision-making and thus not reflect patients’ priorities [6]. Alternatively, it can be argued that heuristic decisions should be respected because heuristics can improve decision-making by allowing patients to integrate complex information that they would otherwise be unable to assimilate [4].

The controversial practice of risk-reducing mastectomy (RRM) focuses this ethical dilemma acutely. RRM reduces breast cancer (BC) risk by surgical removal of breast tissue. It improves life expectancy in patients at high risk, defined as having probable BRCA1/2 or P53 gene mutation [7]. However, although RRM may reduce contralateral BC in lower-risk BC survivors [8,9], it does not change life expectancy [7]. Moreover, RRM is irreversible and exposes healthy patients to iatrogenic risk [10,11]. The incidence of RRM is increasing. In the UK, 600 patients received bilateral RRM in 2002 and 1,186 in2011 [12]. In the USA, the use of contralateral RRM tripled from 3.9% of women treated by mastectomy for BC in 2002 to 12.7% in 2012 [13].

Curiously, the growth in RRM is insensitive to objective risk [13,14]–that is, it has grown even in patients for whom benefit is questionable. There is evidence that its use is driven by patient requests. One UK centre reported a ‘spike’ in RRM following publicity about celebrities who had chosen RRM [15] and Beesley et al. [16] found, in a sample of 60patientsreceiving RRM in a different UK centre, that nearly all had initiated discussion of RRM with their surgeons. Evidence also suggests that many patients request RRM because they use an emotional decision heuristic; that is, their RRM decisions reflect their own worry about BC rather than rational weighing of risks and benefits [17,18]. However, little is known about how emotions or other heuristics influence patients’ decision-making, and whether this distorts or improves decisions.

The final decision for RRM is made by breast surgeons. Although surgeons have guidance about risks and benefits of RRM for different patients [19], there is no guidance for how they should evaluate and respond to patients’ preferences. A pre-requisite for developing such guidance is to understand how patients make their decisions. This was the aim of the present study. In order to avoid making *a priori* assumptions about patients’ decision processes, we used qualitative methods to explore their decision-making inductively. We aimed: (i) to describe how patients decided whether or not to seek RRM; and (ii) to identify implications for how surgeons should respond to these decisions. In addressing these aims, we also sought to inform broader debate about how clinicians should respond to patient decisions that are made heuristically rather than rationally.

## Method

### Participants

From October 2013 to March 2015, we recruited patients who had considered whether or not to have RRM, had made their decision to have or not have RRM and had informed the research team that they did not plan to revisit that decision. Patients were sampled from a specialist breast surgery unit in a teaching hospital serving a socio-demographically mixed urban area. In this unit, five surgeons (three female) routinely provided RRM for patients who were at increased risk because of family history in first-degree relatives (usually with confirmed BRCA1/2 or other genetic mutations) or because they had already experienced BC. Surgeons also considered RRM for patients whom surgeons believed were highly distressed about possible BC and for whom alternative prevention or surveillance strategies were not feasible. Bilateral RRM (BRRM) is mastectomy of both breasts, either in patients who have not experienced BC or who have previously been treated for BC with breast conserving surgery. Contralateral RRM (CRRM) is mastectomy of the opposite breast in patients who have already had one removed, usually after treatment for BC. BRRM surgery was performed after patients had discussed the risks and benefits with surgeons and a multidisciplinary team, CRRM after discussion with a surgeon. All patients had a consultation with a clinical psychologist before confirming their decision.

To ensure that we obtained a wide range of experiences of decision-making, purposive sampling was used to include both BRRM and CRRM, patients who had and had not experienced BC, patients with and without known BRCA1/2 mutation and/or family histories of cancer and patients who had opted and not opted for surgery. We also asked all staff on the unit to alert us to patients who had considered but not requested RRM. Staff failed to recall such instances in patients who had experienced BC. However, we were able to recruit two patients, without BC or identified gene mutations but with family BC histories, who both decided not to accept the offer of BRRM (P24 and P25). Patients were interviewed as soon as possible after surgeons listed them for surgery. The two who did not opt for surgery were interviewed within two weeks of being referred to us.

### Procedure

The study was approved by the North-West England Research Ethics Service (13/NW/0421). Members of the clinical team (surgeons, nurses, genetic counsellors or psychologists) identified potential participants before scheduled appointments at routine clinics, explained the purpose of the project and referred interested patients to a (female) researcher present in the clinic. The researcher gave written details of the study and offered patients a one-week ‘cool off’ period before interview to consider their decision to participate. A week later, the researcher gained patients’ written consent and performed a semi-structured face-to-face interview in their homes or in a private room in the hospital as patients preferred. Interviewers were HGF (a Clinical Psychologist in training with experience of clinical interviewing) and PSaini (PhD in Psychology and trained and experienced qualitative interviewer). Field notes were made after interviews. The interviewers used open questions, prompts and reflection to achieve a conversational style. The research aim provided an initial direction to an interview guide, which evolved in response to the developing analysis. Broadly, all interviews covered: a timeline of key clinical events and associated transitions in knowledge, expectations and attitudes to RRM; how patients made decisions; thoughts and feelings associated with the decision; perceived risks and benefits of RRM; whether and how other people, including clinical staff as well as family, friends and social contacts, influenced decisions; and how and why patients presented their decisions to other people. Interviewers pursued any content of interest to the research aim that did not appear in the interview guide. Interviews were digitally audio-recorded with participants’ permission, then transcribed pseudo-anonymously.

### Data analysis

Analysis was performed in parallel with data collection. Transcripts were read by SLB, HGF, P Saini and P Salmon to allow inductive interpretation of the accounts, incrementally forming a thematic framework which was tested and modified by ‘cycling’ between the developing analysis and new data. The emerging analysis was recorded as an evolving document and discussed regularly amongst all authors. Analysis was finalised when theoretical saturation was reached. Our interviews carry the danger that pre-decisional processes are mis-remembered, reconstructed in the light of the decisions, or ‘altered’ to fit a preferred interpretation [20]. We were also alert to the tendency of interviews about contentious or emotive topics to elicit justifications rather than explanations for behaviour. Therefore, we did not regard interviews as providing direct access to participants’ experiences and intentions, but interpreted them in the context of the whole interview, other interviews and field notes. Each transcript was read by at least three authors, the whole team providing a broader reference group which reviewed and tested the developing analysis. Consensus validity was ensured by discussing the analysis within the team [21]. Where analysts disagreed, points of disagreement were noted and resolved by discussion and review [21,22]. Reflexive validity [21] was achieved by recording the extent to which conceptual categories developed and changed during analysis. Other events recorded were insights that guided the development of the analysis, and pivotal cases that challenged the emerging analysis. Standards by which the developing analysis was assessed included catalytic and theoretical validity, by which we mean, respectively, that findings should have the potential to change practice for the population being studied, and that they should add to existing theory.Key findings are illustrated by italicised quotes, with ellipses (…) indicating omitted text and explanatory comments in square brackets. Participants’ study numbers and clinical backgrounds (BC or not, gene mutation or not) are indicated for each quotation.

## Results

### Composition of sample

Of 38 patients who met the researcher, all consented. We were unable to contact two to arrange interviews, so the final sample was 36 patients. Twenty-two were BC survivors, of whom six had gene mutations. Of the 14 non BC survivors, nine had confirmed gene mutations. Of the remaining five, all were considered high risk of BC due to family history of BC or personal histories of ovarian cancer. One had a negative result for BRCA but was being tested for other mutations, two had not been tested, one had been tested but the result was inconclusive and one was awaiting her test result. Table 1 provides demographic and clinical details for each participant.

**Table 1:** Sample characteristics.

ID	Age	Breast Cancer history	Time since diagnosis	Family Breast Cancer history	Genetic testing status	RRM	Highest education level	Work status	Job type
1	46–50	Yes	25 months	No	No test	CRRM	Diploma	Employed	Managerial
2	35–40	No		Yes	Inconclusive	BRRM	School	Employed	Clerical
3	60–65	Yes	20 months	Yes	BRCA1/2	BRRM	School	Unemployed due to illness	Office
4	25–30	Yes	12 months	No	BRCA1/2	CRRM	Vocational qualification	Employed	Skilled manual
5	46–50	Yes	20 months	Yes	Waiting for test	CRRM	School	Sick leave	Professional
6	46–50	Yes	12 months	No	BRCA1/2	CRRM	School	Sick leave	Unknown
7	56–60	Yes	12 months	Yes	BRCA1/2	CRRM	Vocational qualification	Employed	Clerical
8	46–50	Yes	34 months	yes	BRCA1/2	BRRM	School	Unemployed	Clerical
9	46–50	Yes	24 months	No	No test	CRRM	Degree	Employed	Clerical
10	46–50	Yes	84 months	no	No test	CRRM	School	Employed	Office
11	31–35	Yes	18 months	Yes	Inconclusive–further testing	CRRM	School	Self- employed	Managerial
12	46–50	Yes	7 months	Yes	No test	CRRM	Degree	Employed	Professional
13	51–55	Yes	5 months	No	Inconclusive	CRRM	School	Employed	Professional
14	46–50	No		Yes	BRCA1/2	BRRM	Vocational qualification	Employed	Professional
15	41–45	Yes	9 months	No	BRCA1/2	CRRM	Degree	Employed	Professional
16	26–30	No		Yes	BRCA1/2	BRRM	Vocational qualification	Employed	Clerical
17	46–50	No		Yes	No test	BRRM	School	Sick leave	Professional
18	51–55	Yes	9 months	Yes	No test	CRRM	School	Employed	Clerical
19	36–40	No		yes	BRCA1/2	BRRM	School	Employed	Managerial
20	66–70	Yes	16 months	Yes	No test	CRRM	School	Employed	Professional
21	26–30	No		Yes	BRCA1/2	BRRM	Degree	Employed	Professional
22	60–64	Yes	9months	Yes	BRCA1/2	BRRM	Degree	Sick leave	Professional
23	41–45	Yes	10 months	yes	Negative	BRRM	School	Part-time	Professional
24	41–45	No		yes	Negative BRCA1/2 –further testing	Decided against BRRM	School	Unemployed due to illness	Professional
25	36–40	No		yes	No test	Decided against BRRM	School	Employed	clerical
26	51–55	No		yes	BRCA1/2	BRRM	PG degree	Employed	Professional
27	31–35	No		yes	BRCA1/2	BRRM	Vocational qualification	Employed	Professional
28	46–50	Yes	42 months	yes	No test	CRRM	Degree	Employed	Professional
29	41–45	Yes	7 months	yes	Negative BRCA1/2 –further testing	BRRM	School	Employed	Professional
30	46–50	Yes	60 months	no	Negative	CRRM	Vocational qualification	Employed	Clerical
31	51–55	Yes	6 months	no	No test	CRRM	School	Employed	Clerical
32	41–45	No		no	BRCA1/2	BRRM	School	Unemployed	Housewife
33	46–50	No		yes	Awaiting outcome	BRRM	School	Employed	Professional
34	46–50	Yes		yes	BRCA1/2	BRRM	Degree	Employed	Professional
35	31–35	No		yes	BRCA1/2	BRRM	Vocational qualification	Employed	Clerical
36	31–35	No		yes	BRCA1/2	BRRM	Degree	Employed	Professional

### RRM was generally a ‘no-brainer’

Some patients wanted to restore symmetry after BC treatment mastectomies. However, the overwhelming reason cited by all patients for choosing RRM was that they feared and felt vulnerable to BC and that RRM offered them safety from BC. Choosing RRM was therefore largely obvious to them, unless they had countervailing fears of the procedure itself. Several used the term ‘*no brainer’* to describe a ‘decision’ that barely required consideration. Many patients did deliberate about the decision and consulted with others–but they did so only after they had made their decision to strengthen and justify it.

Where we detected differences in decision-making between groups of patients they concerned the balance of fears of BC and RRM. As we describe below, some patients without BC experienced their vulnerability with less emotional intensity than those with BC, and some patients had countervailing fears of RRM. Consequently, the decisions for these groups felt less obvious. We saw no systematic differences between women with and without BRCA1/2 mutations.

### Decision-making was dominated by fear and vulnerability

Patients generally did not find probabilistic estimates of risk relevant; ‘*I think when you start getting into statistics and percentages it*, *it becomes a bit of a game*, *doesn’t it*, *you know?*’(P15: BC, no gene mutation). P9 (BC, no gene test) described explicitly the dissociation between objective and subjective risk; believing her objective risk of BC to be ‘*5–10%*’, she described her decision to opt for RRM as a response to a feeling ‘*in my head’* that risk was ‘*about 80*%’.

Instead of trying to estimate objective probability, patients felt a sense of vulnerability that felt ‘unbearable’ and that precluded ‘normal’ life. Those who had experienced BC felt this vulnerability acutely, describing an immediate and almost visceral sense of menace—an implacable enemy that hides like a *‘time bomb’* inside their bodies; *‘She* [doctor] *just said “The type of cancer you had*, *you wouldn’t have felt it* [a cancer detected by screening]”. *So*, *that’s when the brain started ticking thinking “so I might have it and not even know then*, *again”*. *So it’s tormented me*’ (P10: BC, no gene mutation). Difficulties in detection or diagnosis compounded the sense of menace: ‘*Yeah*, *just not being able to ever detect it*, *that is my main worry*, *that they could literally monitor the other one* [breast] *now for the rest of my life and still not find it*. *Because it has happened*, *it’s actually*…*If something that horrible has happened to you*, *you sort of don’t trust the* [diagnostic] *tests anymore*.’ (P5, BC, no gene test). Many patients made immediate decisions, often asking for RRM upon diagnosis, even before they knew of it as a clinical option; ‘*I asked for it* [BRRM] *as soon as I knew I was having one mastectomy*. *I asked to have them both done at the same time*; (P11: BC, gene test inconclusive).

Patients with no history of BC also spoke of subjective vulnerability rather than objective risk. However, many used less emotional language, typically referring to a more abstract state of being ‘*at risk*’ or ‘*at high risk’*, and *‘reducing risk’* through RRM; *‘It’s enough for me to know that* [RRM] *substantially reduces the risk of me getting breast cancer in my left breast*.*’* (P15: no BC, no gene mutation). Consistent with their less emotive language, there was less urgency about RRM in these patients. Several did not make immediate decisions and two decided against surgery (P24: no BC, gene test pending; P25: No BC, no gene test).

For five patients (including ones with and without history of BC), fears of surgery (particularly of dying under anaesthesia) were a counterweight to their fears of BC and they found it hard to decide. Three (P20; BC, no gene test; P24: BC, gene test negative and P25: no BC, gene test negative) eventually chose RRM, but after varying periods of what they generally portrayed as indecision. P20 deliberated for several months before making her decision as she wavered between being directed by her fears of BC and her fears of surgery; ‘*I’ve weighed it up and changed my mind again and again and again*, *and then I’ve just come down on the side of “I think I’ll get it done*, *yeah’*.

### Patients felt RRM to be applicable to them

All patients appreciated the seriousness of RRM, several describing it as ‘*drastic’* or a ‘*mutilation’*. Nonetheless, none reported having questioned whether RRM was appropriate for them in principle. Several had known that RRM was sometimes performed but had not considered it for themselves until they encountered surgeons, friends, family or other patients who advised RRM or who simply indicated that it was an option. These patients reported that others influenced them by making benefits of RRM seem applicable to them personally; *‘And then after the surgery* [mastectomy] *and during chemotherapy … a lady got talking to me in* [a supermarket] *and she just said “Oh have you got breast cancer?” And I said “Yes” … She said “I’ve just been to see my consultant actually to get the other one removed”*. *And I was standing there thinking “Do you know*, *that’s what I need to do*.*”‘* (P1: BC, no gene test).

### Doing ‘all I can’ to feel safe

Patients frequently cited wanting to feel ‘safe’, but safety did not mean freedom from objective risk of BC. Safety meant reducing their sense of vulnerability to BC and the intense fear associated with that vulnerability: ‘It’s [RRM] to benefit my mental health in the future, to reduce the worry in the future because… if you’re checking and you feel something slightly lumpy … you’re going to be stressed out to death, until it gets sorted.’ (P11: BC, gene test inconclusive). Patients knew that they could not completely eliminate risk. Most explicitly acknowledged the residual risk of local recurrence or new BC, although they wanted as much tissue removed as possible to minimise this. Some were explicit that RRM cannot reduce metastatic risk. In general, however, patients did not distinguish between new cancer and distant recurrence.

Safety arose, instead, from a sense of having done ‘all I can’ or ‘all in my power’ to prevent BC. For them, doing ‘all I can’ to eliminate preventable risk was sufficient; ‘God forbid, if it does come back, well that’s something I’ve got to … deal with then, when it happens, if it happens. So but it’s still, I’d still know in my heart of hearts that I’d done everything I can do, you know” (P7: BC, gene test positive). That is, choosing RRM avoided future regret; “if it does come back and I didn’t do something about it when I could have done” (P10: BC, no gene test).

Choosing RRM was the only action that patients cited spontaneously when describing the importance of doing ‘*all I can’*. When prompted, some had gained a sense of safety from chemoprevention programmes. For example, P10 described her ‘*tamoxifen* [hormone treatment] *blanket*’, and opted for RRM only when her tamoxifen programme ended. However, for most patients, RRM was unquestioned as the obvious and only act that they could initiate to achieve safety.

### Where ‘deliberation’ did not occur before the decision, it often occurred after

About half the participants who made immediate decisions gave accounts resembling deliberation, but this occurred *after* they had resolved to undergo RRM. These patients did not question their decision and none changed it. Instead, they generated arguments that supported their decisions. P9 (BC, no gene test) was explicit that this post-decisional process was a way to ‘*rationalise’* a decision that she felt had been made ‘*emotionally’*; ‘*It was an unusual… way to make a decision for me*, *but it was the emotion made the decision*, *the moving it to the practical … just*, *I think*, *helped me rationalise it… and helped me make myself feel comfortable with an emotional decision’*. Similarly, P11 (BC, gene test inconclusive) described the importance of this process for being comfortable with the decision she had already made; ‘*I kind of always knew*, *I just knew I had to go through this whole process of weighing everything up … And as you’re*, *you know*, *going through all the whole ups*, *pros and cons of everything it’s quite a personal thing*, *that*, *I think*, *and I don’t think anyone can really help you on that one’*.

This process of deliberation included rehearsing the risk-reducing benefits of RRM and identifying other reasons in its favour, such as achieving body symmetry. It also included consulting with friends, family and clinical staff, whereby patients generally sought not to test their decision but to enlist others’ validation or approval for it. Two (P1: BC, no gene test, P15: BC, no gene mutation) explicitly indicated that they wanted approval. P15 stated “*I’m looking for them to say that it’s a good idea’*. Patients were disappointed when endorsement was withheld. P16 (no BC, gene test positive) became upset during her interview when she explained that friends and family ‘*do not understand’* her decision. Others wanted surgeons to be enthusiastic about their choice, and became annoyed or upset when they felt that surgeons were not. Although most trusted their surgeons’ opinions and claimed to take them into account, they persisted with their decisions even when they felt that surgeons lacked enthusiasm as P13 (BC, gene test inconclusive) illustrates; ‘*I feel that the clinical team have a perception which is*, *I think*, *purely based on clinical risk*, *and I don’t think that their interpretation of*, *of that risk should be the only thing that they use… So in a way I think they should keep their opinions to themselves*, *because it isn’t a pure clinical issue*…*And I suppose I was quite taken aback at the sort of negativity that was attached to a decision that I*, *you know*, *that I wanted*.*’*

## Discussion

Although most patients described seeking RRM to reduce risk, they did not generally consider objective risks and benefits and, indeed, regarded these as irrelevant to their decision. Decisions were, instead, shaped by fears of BC and of not having done ‘all they could’ to prevent it and, for some patients, countervailing fears of RRM surgery itself. Fear activated an emotional decision-making heuristic; patients wanted to feel safe from their most salient fears. Their decisions therefore reflected a ‘balance of terrors’: those associated with BC on one hand and surgery on the other. For most patients, fear of BC outweighed that of RRM and decisions were ‘obvious’ and easy. Having done ‘all I can’ defined the sense of safety that patients sought by choosing RRM, whilst tolerating local and distant risk that RRM could not prevent. Decisions were more difficult for patients whose fear of BC was counterbalanced by fears of surgery or its consequences, because no decision offered safety.

These findings are, at first sight, consistent with previous suggestions that patients use emotion as a heuristic or ‘short-cut’ in decision-making about RRM, and that this heuristic assumes primacy over consideration of objective risks and benefits [17,18]. However, existing theoretical accounts of heuristic decision-making describe people using emotion as a proxy for objective risk [23] or as a warning of vulnerability [24]. For our participants, fear influenced their decisions in an additional way. Fear reduction became the primary decision-making goal.

Although patients rarely deliberated about RRM before deciding that they wanted it, many did so afterwards. That is, they engaged in extensive post-decisional reasoning and consultation with others. They did not revisit their decision; no woman changed her decision, and consultation was more about enlisting support than engaging others’ views. Instead, post-decisional deliberation was biased to defend their chosen position rather than test its validity, and recruitment of other views was biased to endorse the decision [25].

Our findings are problematic from the perspective of current normative views of medical decision-making. In an influential account, Elwyn and Myron-Shatz [26] describe three essential characteristics of good decision-making: patients should understand possible options and the potential consequences of these options; they should appreciate the potential personal significance of these consequences; and they should consider this significance when making decisions. Decisions in the present study did not meet these criteria. Patients made decisions for emotional reasons and many did not consider the possible consequences and implications until later.

Instead, the findings can be understood from the perspective of decision-making theories that consider the psychological functions that decision-making fulfils [27,28,29]. In particular, Svenson’s ‘differentiation and consolidation’ theory [27] states that decision-making has two linked functions: solving the decision problem, whilst ensuring that individuals are prepared psychologically for threats that they might experience to their choice in future. That is, people strive to minimise potential regret associated with having made a ‘wrong’ decision. The latter function underlies two crucial elements of Svenson’s theory. First, people seek solutions that are ‘differentiated’; that is, appear sufficiently superior to others to minimise the potential for regret. Second, people engage in ‘consolidation’, a post-decision deliberation process aimed at reducing any potential for regret by strengthening confidence in the initial decision. The latter process is typically biased because it emphasises evidence or views that support the decision [30].

For the minority of patients in our study whose fear of BC was counterweighed by that of RRM there was no clearly differentiated decision. They considered alternatives and found decision-making difficult. For most, however, the heuristics of fear-reduction and doing all they could to prevent BC pointed to RRM as an option that was sufficiently differentiated that they did not need to consider alternatives. Thus, RRM promised freedom from fear and protection from potential regret. These patients did, however, face a profound psychological threat to being content with their decision. Choosing RRM was one of the most significant decisions in their lives, and was made in a clinical and cultural context that expects big decisions to be made rationally and in consultation with others. Because the decision had been largely emotional and solitary these patients needed, as P9 indicated explicitly, to protect themselves from potential future regret at having made a poor decision [31]. The process of post-decision deliberation that many of these participants recounted therefore functioned as the consolidation process that Svenson described.

This study aimed to understand why patients opted for RRM, but we have fewer insights about why they did not. Clinical staff struggled to find cases where patients contemplated RRM, but decided against it. The failure to find such cases may, in fact, illustrate our finding that patients who contemplate RRM are then very unlikely to reject it. Cases were sampled from a single unit, and, thus, patients’ views may be influenced by policies and practices within the unit that are not necessarily common to all units.

Our findings point to two ethical issues confronting surgeons who offer RRM, concerning *why* and *how* patients chose the procedure.

Patients were clear about *why* they wanted RRM. Therefore, from the perspective of normative expectations on clinicians to respect patients’ own priorities [32], patients made decisions freely and consistent with a personal goal that, for most, outweighed other considerations. They wanted to be free of fears of BC and to know they had done all they could to mitigate the risk of BC. RRM surgery can indeed alleviate fears of BC recurrence [17], and it is not clear that other approaches can do so [33]; so surgery is a plausible way to achieve the outcome patients sought. Using a surgical solution, which carries a risk of harm, to achieve a psychological goal is, however, ethically complex, particularly where surgery carries little prospect of survival benefit. Ethical analyses of cosmetic [34] and bariatric surgery [35] have argued that such interventions could be justified, provided that benefits outweigh risks, benefits are likely to occur and benefits cannot be achieved with less risk.

If RRM is to be considered potentially acceptable as a surgical response to a psychological need, its ethical justification would depend on *how* patients make their decisions. However, our findings expose a tension between normative views of how patients should make decisions and psychological theory about how they decide in reality. From the current normative perspective [26], patients’ failure to deliberate about decisions, examine the evidence, and weigh the available options reduced decision quality, and therefore the extent to which surgeons should respect their decisions. Viewed, by contrast, through the psychological lens of differentiation and consolidation theory, patients made decisions in a way that is understandable because it met psychological needs associated with decision-making.

Kleinman [36] warned that ethical guidance about dilemmas in clinical practice risks being unrealistic if it is not grounded in understanding how people normally resolve these dilemmas. That is, evidence about how people ‘are’ has to be the starting point for developing guidance about how people ‘should be’. Our findings illustrate how heuristic reasoning is probably unavoidable where patients are confronted with complex information that they do not have the time, knowledge or emotional distance to weigh objectively [4,5]. Therefore, rather than trying to impose an alien norm of rational decision-making onto RRM decisions, it is more realistic to make patients’ existing heuristic approaches the starting point for considering how clinicians should respond.

In proposing normative criteria for good decision-making, Elwyn & Myron-Shatz [26] conceded that many patients will make decisions heuristically. Thus, the clinician’s task is not to replace that reasoning with a more ‘rational’ mode, but to ensure that patients have considered the range of options and consequences and how they would be affected by these. The Elwyn and Miron-Shatz perspective has different implications for two groups of patients in our study. By definition, the minority of patients who were wrestling with competing fears were already aware–and frightened–of at least two possible outcomes. From the perspective of Elwyn and Myron-Shatz, clinicians’ responsibility to these patients would be to help them understand these outcomes and other possible outcomes that they have not considered.

For most patients, whose fear of not having done all they could to prevent BC led them to choose RRM without deliberating before the decision, and who approached surgeons with decisions already made, our findings on post-decision deliberation suggest a novel approach to reconciling normative expectations with psychological reality. Differentiation and consolidation theory views post-decision deliberation as driven by anticipated threats to the validity of the decision that has been made such as, in the current study, the expectation that important decisions should not be made emotionally, and as a personally directed process biased to support that decision. We suggest that clinicians could recruit this process to guide it and to ensure that patients satisfy normative criteria such as those set out by Elwyn and Myron-Shatz [26]–albeit after they have made their decision. For instance, patients who have requested RRM should be guided to think about other available options such as enhanced screening or chemoprevention, and potential consequences and risks associated with these options. How clinical services can best do this, how fully patients could consider options and consequences, and whether such ‘post-decision deliberation’ would influence the ultimate decision, need to be explored empirically. However, more complete consideration of options and their risks and benefits would, arguably, make for better decisions at least inasmuch as patients were meeting normative expectations (not least, their own) that major treatment decisions should be considered ones [31].

### Conclusion

The inescapable emotionality of a patient’s decision does not mean that it cannot be respected as valid. The corollary is that patients need to be supported to make, or review, these decisions in ways that meet normative expectations [26] while being consistent with the reality of the psychological processes involved in decision-making. General characteristics of RRM decisions are likely to apply to other controversial cancer risk-reducing procedures, such as oophorectomy, prostatectomy or hormonal therapies, which have iatrogenic effects but may be sought by people seeking escape from worry. Indeed, they may apply more broadly to health care decisions where the defining features of patients’ decision-making in the present study are present: fear of a mortal threat, and an invasive or dangerous intervention by which patients feel they can mitigate the threat.

Nonetheless, our findings cannot simply be generalised to these decisions. The immediate lesson is the need for ethical reflection to be based on detailed analysis of how patients approach specific decisions. Our study provides a template for researchers and clinicians to approach dilemmas about how to regard patients’ decisions that are made heuristically. Research of this kind can inform development of normative theory about heuristic decision-making that is workable in clinical practice as well as ethically robust.
